# Characteristic of mental health app usage: a cross-sectional survey in the general population

**DOI:** 10.1186/s12889-024-20500-1

**Published:** 2024-11-12

**Authors:** Sophia Fürtjes, Elisabeth Gebel, Hanna Kische, Katja Beesdo-Baum

**Affiliations:** TUD Dresden University of Technology, Behavioral Epidemiology, Institute of Clinical Psychology and Psychotherapy, Chemnitzer Straße 46, D-01187 Dresden, Germany

**Keywords:** E-mental health, Mental health apps, Survey, General population, App usage

## Abstract

**Background:**

Mental health apps (MHA) have gained popularity in recent years. Most freely available apps are of low quality and lack evidence for effectiveness. Yet, download rates indicate high usage. MHA are potentially beneficial for individuals with no/low symptom severity as prevention, or as an option to reach underserviced populations. However, currently very little is known about the characteristics of MHA users in the general population, about the kind of MHA used, or the motivation behind MHA usage.

**Methods:**

We collected survey-data from *N* = 1,247 individuals from the general population to investigate MHA usage.

**Results:**

Descriptive statistics revealed that 41% had used MHA in the past 12 months; prescription app-use however was low (1.5% of MHA users). Mindfulness, moodtracking, and relaxation were the most popular categories of MHA. Perceived helpfulness, fun, and availability were the top reasons for MHA usage. Non-users reported distrust, lack of perceived need, and data security concerns as usage barriers. MHA users and non-users did not differ regarding sociodemographic variables. Multiple regression analyses revealed that sub-threshold symptoms of psychological disorders as well as higher levels of anxiety and stress were linked to higher probability of MHA usage. Higher levels of depression were associated with lower likelihood of MHA usage in general, but increased likelihood of usage of self-help apps specifically for depression.

**Conclusions:**

It follows that MHA use is common in the general population independent of sociodemographic characteristics. MHA can reach people who might benefit, but more awareness and better structure of the market is needed to reduce distrust, increase usage of high-quality MHA, and improve the fit between app and user.

**Supplementary Information:**

The online version contains supplementary material available at 10.1186/s12889-024-20500-1.

## Background

Although mental health apps (MHA) have received increased attention in research over the last years, no uniform definition can be inferred from the literature. Generally speaking, MHA could be defined as digital applications supported by mobile technologies targeting mental health topics such as depression, anxiety, or stress. A more nuanced perspective could further differentiate between apps targeting specific symptoms or disorders (e.g., anxiety, depression, alcohol addiction etc.) with an intervention-focused approach (*self-help apps*), and apps focused on fostering general wellbeing without specific indication through e.g., mindfulness exercises or meditation (*self-care apps*). In addition to these two groups of apps, a third group could be specified that lies outside the proposed definition of MHA, i.e., does not specifically target mental health topics, but can still be used in a way that supports mental health (*non-MHA apps*). For example, although mood-tracking is not the target of period-apps, it is often an available feature. Or consider Rituals©, a shopping-app for cosmetic products, which also offers meditation exercises. While these are not MHA per se, they could be utilized to improve mental health and wellbeing.

This already hints at difficulties in navigating the market of MHA. The number of MHA available for download is high (even when the last group of apps, *non-MHA*, is disregarded) and the market is very volatile [1, 2]. New apps are made available while others disappear from Apple’s AppStore and Google’s PlayStore in constant flux. Only a minuscule proportion of the available apps has been developed by health care professionals, even less provide evidence regarding effectiveness [3–5]. This lack of empirical foundation does apparently not discourage usage. High download-rates [3] indicate that many people use MHA, even though equally high attrition numbers show that usage is often short-lived [6, 7]. While research on this matter is scarce, the high attrition probably results from different factors, with low effectiveness, lack of quality, and suboptimal fit between app and user most likely being among them.

For those MHA that have been empirically evaluated, results show that self-help MHA can be effective in e.g., reducing symptoms of depression or anxiety, improving quality of life, or increasing positive affect [8–10]. However, while reviews have shown that MHA can potentially be beneficial [2], the vast majority of publications regarding this topic to date have low scientific quality [11]. The scarce available evidence suggests that self-help MHA might primarily be beneficial for people with low symptom severity, as preventative interventions, or as a supplement to treatment [12, 13]. They could also reach underserviced populations, circumvent barriers such as stigma, or bridge time spent waiting for therapy [13, 14].

Potential “target populations” for self-help MHA would therefore be individuals with impaired mental health with low symptom severity, individuals at risk of developing mental health problems, or individuals waiting for treatment. Experts have recommended integrating MHA into the public health care system via payment channels similar to medication [15, 16]. Sufficiently validated MHA could be prescribed to individuals falling into the categories mentioned above (low symptom severity, high individual risk, etc.), and integrated into primary care. The public health care system in Germany has adopted this approach for self-help MHA in 2020 by introducing Digital Health Applications (DiGAs), which can be prescribed by general practitioners, psychiatrists, or psychotherapists and are covered by insurance. Currently 24 MHA are available for prescription, most of them target depression or anxiety (https://diga.bfarm.de/de/verzeichnis).

The “target population” for self-care MHA is harder to determine. These MHA, e.g., meditation apps etc., could be beneficial for healthy individuals to maintain their mental wellbeing. They could also serve individuals with slight impairments in mental wellbeing below the threshold of mental health problems. Lastly, they could also be used as a supplement to self-help MHA or treatment - in this case, the target population for self-care MHA would overlap with the target population for self-help MHA. However, self-care MHA are not included in the prescription-based DiGA-system described above. Some insurance companies provide their own self-care MHA or digital prevention-classes as an addition to traditional prevention-classes (which are also (partly) covered by insurance). Nevertheless, there is no official place for self-care MHA within the public health care system. It is unclear whether potential target populations are actually being reached, both by freely available MHA and by prescription-based MHA. To our knowledge, no research exists to date on the characteristics of MHA users. While this population appears to be growing (as indicated by increasing download-rates), we do not know who actually uses MHA – and who does not and why. What kind of MHA are most commonly used vs. are less popular is also difficult to know given the highly volatile market, inconclusive data on download rates and high attrition. The motivation for usage vs. non-usage is also understudied, although some preliminary research points into the direction of a high general willingness to use MHA in the general population [17]. In general, self-help is frequently used especially within the range from low, sub-clinical up to moderate, clinically relevant symptom severity, even though it is not always considered to be helpful [18, 19]. One might therefore speculate that MHA as a form of self-help are frequently used due to their availability and low cost, but usage might quickly be discontinued due to lack of experienced helpfulness. Further aspects such as unattractive payment-schedules, low motivation, or a bad fit between app and individual might also contribute to attrition.

The current study aims to provide information on the current state of MHA usage in the general population in Germany, the characteristics of MHA users, and the reasons for usage as well as barriers to (continued) MHA use. The research questions are:

1. What is the frequency of MHA usage in the general population in Germany and what kind of MHA are being used?
2. What are the reasons for usage, discontinuation of usage, and barriers hindering usage of MHA?
3. What are the sociodemographic characteristics and the mental health status of MHA users vs. non-users?

The data will provide information on whether “typical” MHA users vs. non-users can be identified. It might also offer preliminary insight in whether potential target groups are currently being reached.

Since this is exploratory, observational research into a new aspect of health care, we refrain from the proposal of specific hypotheses for these questions.

## Methods

### Study design, sample, and procedure

Data was collected between 09/2022 and 03/2023 via a cross-sectional online survey in a convenient sample from the general population in Germany. Participants were recruited via social media, survey platforms, and the crowdsourcing platform Clickworker. Inclusion criteria were age 16 and older, ownership of a smartphone, and sufficient German language skills. *N* = 1,711 participants gave informed consent and participated in the study. We excluded participants from analysis who immediately dropped out after the sociodemographic questions (*n* = 356), who had technical difficulties filling out the survey (*n* = 41), or who had filled out the survey with obvious lack of diligence (*n* = 51; identifiable through impossibly short completion-time and/or contradictory statements in the assessment of sociodemographic variables). The final sample size therefore was *N* = 1,263. Individuals who participated via Clickworker were compensated with 2.77€.

### Measures

Sociodemographic characteristics (age, gender, education, employment, income, living area) were assessed using adapted questions from section A of the DIA-X-5, which is a standardized interview to assess mental disorders [20].

Because to our knowledge, no established measure for usage of MHA exists, this was assessed via a self-developed questionnaire. To avoid overly complicated questions and keep the focus on the main aspects of MHA usage, we decided to assess the number and kind of used MHA, the frequency of usage, and facilitators and barriers of usage. Participants initially gave information on the number of MHA they had used in the past 12 months. Participants who had used at least one MHA were asked about the kind of MHA, selected from eight self-help categories *moodtracker*, *depression*, *anxiety*, *alcohol/drugs*, *eating disorders*, *chronic pain*, *sleeping disorders*, *other*, and three self-care-categories *meditation/mindfulness*, *relaxation*, *motivation/inspiration*, and the last category *other*. These categories were chosen based on the most prevalent mental health problems (depression, anxiety etc [21]). and the categories most often reported in previous literature (mediation, inspiration [3]). For each category in which MHA usage was affirmed, the frequency of use was assessed. Reasons for usage, reasons for discontinuation of usage, and reasons against usage were assessed via multiple selection and optional free text input. Answer categories followed the most common facilitators and barriers of service usage found in the literature [22, 23]. Participants who had never used MHA were asked about their reasons against usage. Lastly, to enable a deeper, qualitative exploration, all participants could give free text input on requests they would have for MHA or circumstances under which they would consider using MHA.

Mental health was assessed both categorically and dimensionally. The adapted research version of the Composite International Diagnostic Screener (CID-5-S; [24] was used to screen for 19 different psychological disorders via 26 items oriented on the stem screening questions of the DIA-X-5 [20]. Questions refer to the time frame of the past 12 months and are supplemented with questions on impairment and/or help seeking in case of an affirmative answer. In the current work, a categorical variable was generated with the three levels “none” (no symptoms affirmed; coded 0), “disorder possible” (symptoms of at least one psychological disorder affirmed; coded 1), and “disorder probable” (symptoms of at least one psychological disorder affirmed and impairment or treatment affirmed; coded 2). Prior work has shown adequate reliability for the CID-S [24] and stem screening questions of the DIA-X-5 [20]. For dimensional assessment of mental health, depression and anxiety symptoms were measured via the 8 item short versions of the Patient Reported Outcome Measurement Information System (PROMIS; [25] scales. Answers are given on a scale from one (*never*) to five (*always*), severity is judged via the mean score. Subjective stress was measured via the short version of the Trier Inventory for Chronic Stress (TICS; [26], consisting of 12 items that are answered on a scale from zero (*never*) to four (*very often*). Chronic stress is judged by the sum score.

## Statistical analyses

### MHA usage

Data on MHA usage and sociodemographic variables were evaluated via descriptive statistics to provide a characteristic of MHA usage. Free-text input regarding the specific names of used MHA was checked for matching between the reported apps and the provided categories. Entries that fit the category, but were non-MHA, were marked as such. Entries that were apps which cannot be used in a way to improve psychological wellbeing (e.g. TikTok etc.) were excluded from analyses. This applied to 98 entries of 66 subjects.

### Associations with mental health

To further investigate whether target populations are currently being reached by MHA, multiple logistic regression was employed to investigate associations between MHA usage within the last 12 months (outcome, coded yes/no) and mental health. We conducted analyses with three binary outcome variables (coded ves/no): usage of any MHA, usage of self-help MHA, usage of self-care MHA. For each outcome, in a first model MHA usage was predicted via the CID-5-S score. In the second model, standardized scores for depression, anxiety, and stress (PROMIS, TICS) were entered as predictors for each outcome. For a more detailed investigation of individual domains in additional models, depression (PROMIS, standardized) was used to specifically predict usage of self-help apps for depression (coded yes/no) and moodtrackers (coded yes/no), and anxiety (PROMIS, standardized) was used to specifically predict usage of self-help apps for anxiety (coded yes/no). All models were adjusted for age, sex, and education.

All analyses were conducted with survey weights. The sample was divided into twelve strata according to age and sex, weights were calculated to achieve representativeness of the general population in Germany [27]. Individuals who had reported a diverse gender (i.e., neither male nor female) were excluded from analyses because weights could not be calculated for these individuals (*n* = 16).

**Fig. 1:**
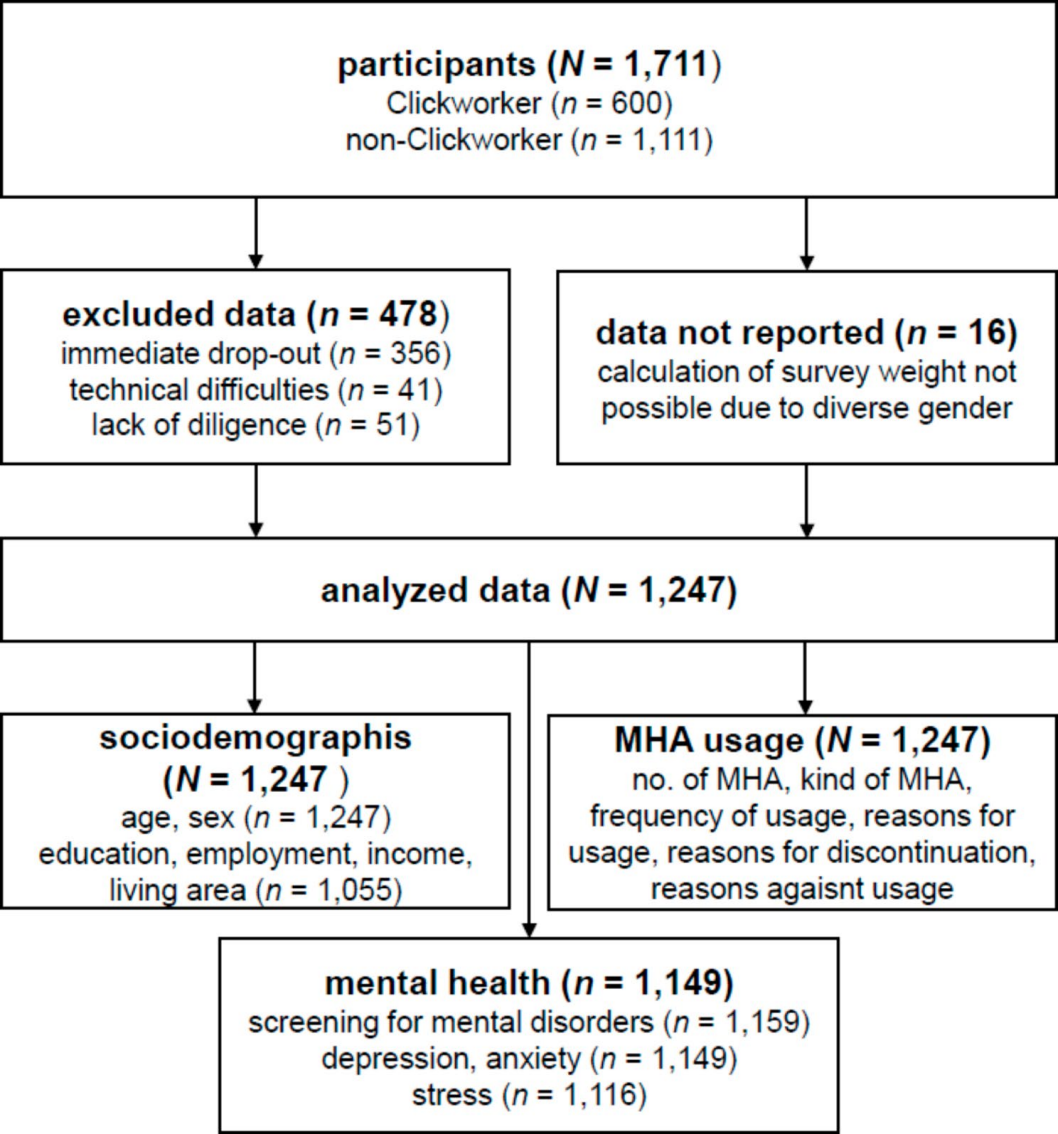
Overview of data flow, available data and analyzed data

Missing data due to drop-out before completion of the survey was not imputed due to the exploratory character of the study. Figure 1 provides an overview of the available data for all measures.

## Results

### MHA usage

Of the 1,247 participants, 41.1%w (weighted; *n* = 504) had used at least one MHA within the last twelve months. Most of those had used one (*n* = 303, 23.4%w) or two (*n* = 128, 12.7%w) MHA, only few participants reported usage of more than two MHA. As can be seen in Table 1, meditation/mindfulness was the most popular category, followed by moodtrackers and relaxation apps. The highest percentage of non-MHA was reported in the category self-help apps for sleeping disorders (e.g., sleep-tracking apps which do not specifically provide information on sleeping disorders or other interventions), moodtrackers (e.g., period apps which also allow mood-tracking), and motivation/inspiration apps (e.g., astrology apps). Apps were used once per week or several times a week by most of the users (*n* = 297, 60.0%w of users), a smaller proportion reported daily usage (*n* = 75, 14.4%w of users). Of the MHA users, 41.9%w (*n* = 198) used both self-help MHA and self-care MHA, 29.5%w (*n* = 135) used only self-help MHA, and 27.1%w (*n* = 104) used only self-care MHA.

**Table 1:** Reported usage of MHA by category

	**no. of users** **(*N*** **= 1,247)**	non-MHA entries	excluded entries
	*n* (%w - col.)	*n* (% row)	*n* (% row)
*self-help apps*:			
moodtracker self-help depression self-help anxiety self-help alcohol/drugs self-help eating disorders self-help chronic pain self-help sleeping disorders self-help other	**195 (13.7)** 97 (7.6) 87 (7.0) 49 (4.1) 43 (3.4) 65 (7.1) 136 (11.5) 100 (8.4)	**18 (9.2)** 4 (4.1) 4 (4.6) 1 (2.0) 1 (2.3) 2 (3.1) **12 (8.8)** 6 (6.0)	7 (3.5) 8 (7.6) 4 (4.4) 2 (3.9) **11 (20.4)** 3 (4.4) 7 (4.9) 2 (2.0)
*self-care apps*:			
meditation/mindfulness relaxation motivation/inspiration	**242 (18.8)** **131 (13.1)** 119 (8.5)	7 (2.9) 6 (4.6) **9 (7.6)**	12 (4.7) 10 (7.1) **22 (15.6)**
*other*	91 (8.8)	4 (4.4)	**10 (9.9)**

*Notes.* MHA = mental health app. Percentages in the first column are the weighted proportion of all participants (%w). Percentages in the second column are the unweighted proportion of non-MHA entries per category. Percentages in the last column are unweighted proportions of excluded entries per category. For each column, the top categories are marked bold.

**Fig. 2:**
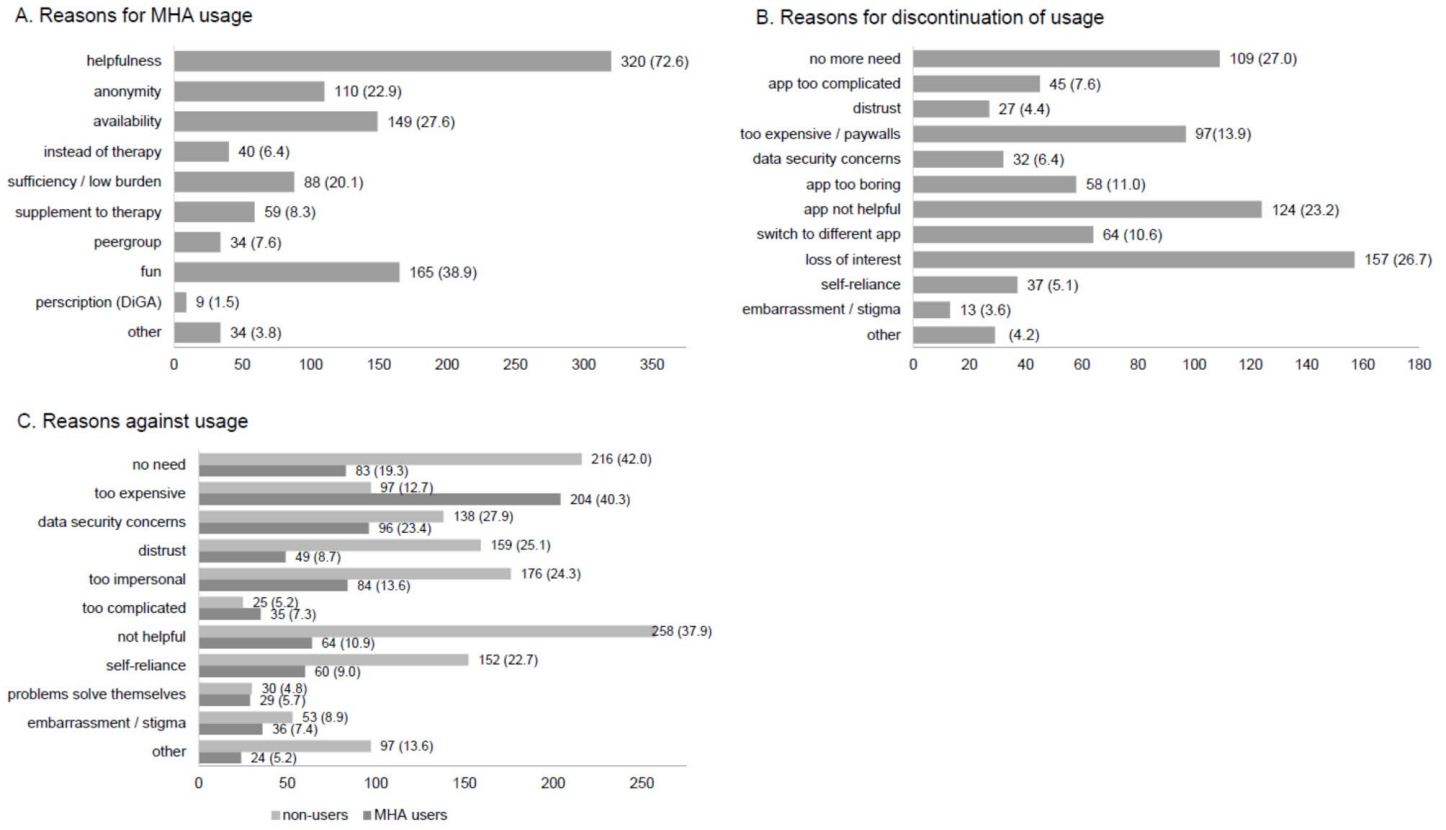
**(A)** Reasons for MHA usage [*n* (%w)], *n* = 504 MHA users. **(B)** Reasons for discontinuation [*n* (%w)], *n* = 504 MHA users. **(C)** Reasons against MHA usage [*n* (%w)], *n* = 504 MHA users and *n* = 743 non-users. Multiple selection possible. Percentages are weighted

The most frequently reported reasons for usage of MHA were perceived helpfulness, fun/enjoyment of usage, and availability (see Fig. 2A). With 1.5%w (*n* = 9), prescription was the least reported reason for MHA usage. Discontinuation of usage was reported to be most often due to a decrease in perceived need, loss of interest, and lack of perceived helpfulness (see Fig. 2B). MHA users most frequently indicated that costs, data security concerns, and lack of perceived need were barriers against MHA usage. Non-users reported more barriers than users, with lack of perceived helpfulness, lack of perceived need, data security concerns, distrust, and apps being too impersonal as the most frequently named reasons against usage (see Fig. 2C).

### MHA user characteristics

A comparison of MHA users vs. non-users regarding sociodemographic aspects revealed no significant differences regarding age, gender, education, or income (see Table 2). MHA users were less often unemployed than non-users (20.6%w vs. 33.0%w, χ² = 20.05, *p* = .019). Regarding mental health, MHA users reported higher anxiety (*F* = 5.08, *p* = .024) and stress (*F* = 7.53, *p* = .006) than non-users (see Table 3).

**Table 2:** Sociodemographic characteristics of MHA users and non-users

	MHA users (*n* = 504)	non-users (*n* = 743)	group comparison
*age [M ± SD]*			
16–24 [*n* (%w)] 25–34 [*n* (%w)] 35–44 [*n* (%w)] 45–54 [*n* (%w)] 55–64 [*n* (%w)] 65+ [*n* (%w)]	44.58 ± 14.67 135 (12.9) 196 (16.0) 88 (18.0) 52 (21.7) 26 (18.3) 7 (13.2)	45.50 ± 16.65 212 (13.8) 307 (17.4) 114 (16.3) 50 (14.6) 48 (22.2) 12 (15.7)	*F* = .26, *p* = .608
*gender*			
female [*n* (%w)] male [*n* (%w)]	299 (52.9) 205 (47.1)	436 (47.9) 307 (52.1)	χ² = 2.99, *p* = .309
*education*			
Abitur [*n* (%w)] no Abitur [*n* (%w)]	318 (65.7) 146 (34.3)	430 (64.8) 161 (35.2)	χ² = .10, *p* = .855
*employment*			
employed [*n* (%w)] unemployed [*n* (%w)]	375 (79.5) 89 (20.6)	437 (67.0) 154 (33.0)	**χ² = 20.05**, ***p*** **= .019**
*income*			
< 500€/month [*n* (%w)] 500–1,000€/month [*n* (%w)] 1,000–2,000€/month [*n* (%w)] 2,000–3,500€/month [*n* (%w)] 3,500-5,000€/month [*n* (%w)] > 5,000€/month [*n* (%w)]	20 (2.5) 54 (8.4) 100 (25.5) 156 (37.1) 91 (17.8) 43 (8.7)	24 (4.3) 78 (10.7) 144 (32.5) 169 (26.7) 124 (18.5) 52 (7.4)	χ² = 17.65, *p* = .161
*living area*			
rural [*n* (%w)] town [*n* (%w)] city [*n* (%w)] big city [*n* (%w)]	78 (14.4) 95 (21.8) 130 (24.0) 201 (39.9)	137 (22.2) 105 (16.2) 168 (19.6) 333 (41.9)	**χ² = 17.70**, ***p*** **= .010**

*Notes*. Weighted descriptive statistics. MHA = mental health application. Age is given in years. Abitur = high school level education. %w = weighted percentage. Available data for education, employment, income, and living area from *n* = 464 MHA users and *n* = 591 non-users.

**Table 3:** Mental health characteristics of MHA users and non-users

	MHA users (*n* = 504)	non-users (*n* = 743)	group comparison
*categorical*			
CID-5-S screening: [*n* (%w)]			
no disorder disorder possible disorder probable	76 (21.1) 249 (53.9) 162 (25.0)	91 (21.0) 317 (49.8) 264 (29.2)	χ² = 2.79, *p* = .620
*dimensional*			
PROMIS depression [*M* ± *SD*] PROMIS anxiety [*M* ± *SD*] TICS stress [*M* ± *SD*]	2.32 ± .89 **2.29 ± .83** **16.26 ± 9.70**	2.33 ± .97 **2.12 ± .87** **13.95 ± 10.15**	*F* = .03, *p* = .874 ***F*** **= 5.08**, ***p*** **= .024** ***F*** **= 7.53**, ***p*** **= .006**

*Notes*. Results of weighted group comparison tests. %w = weighted percentage. MHA = mental health application. PROMIS = Patient Reported Outcome Measurement Systems, possible range 1–5, available data from *n* = 487 MHA users and *n* = 662 non-users. TICS = Trier Inventory for Chronic Stress (TICS), possible range 0–48, available data from *n* = 478 MHA users and *n* = 638 non-users. CID-5-S = adapted Composite International Diagnostic – Screener (CID-5-S), available data from *n* = 487 MHA users and *n* = 672 non-users.

### Associations with mental health

**Fig. 3:**
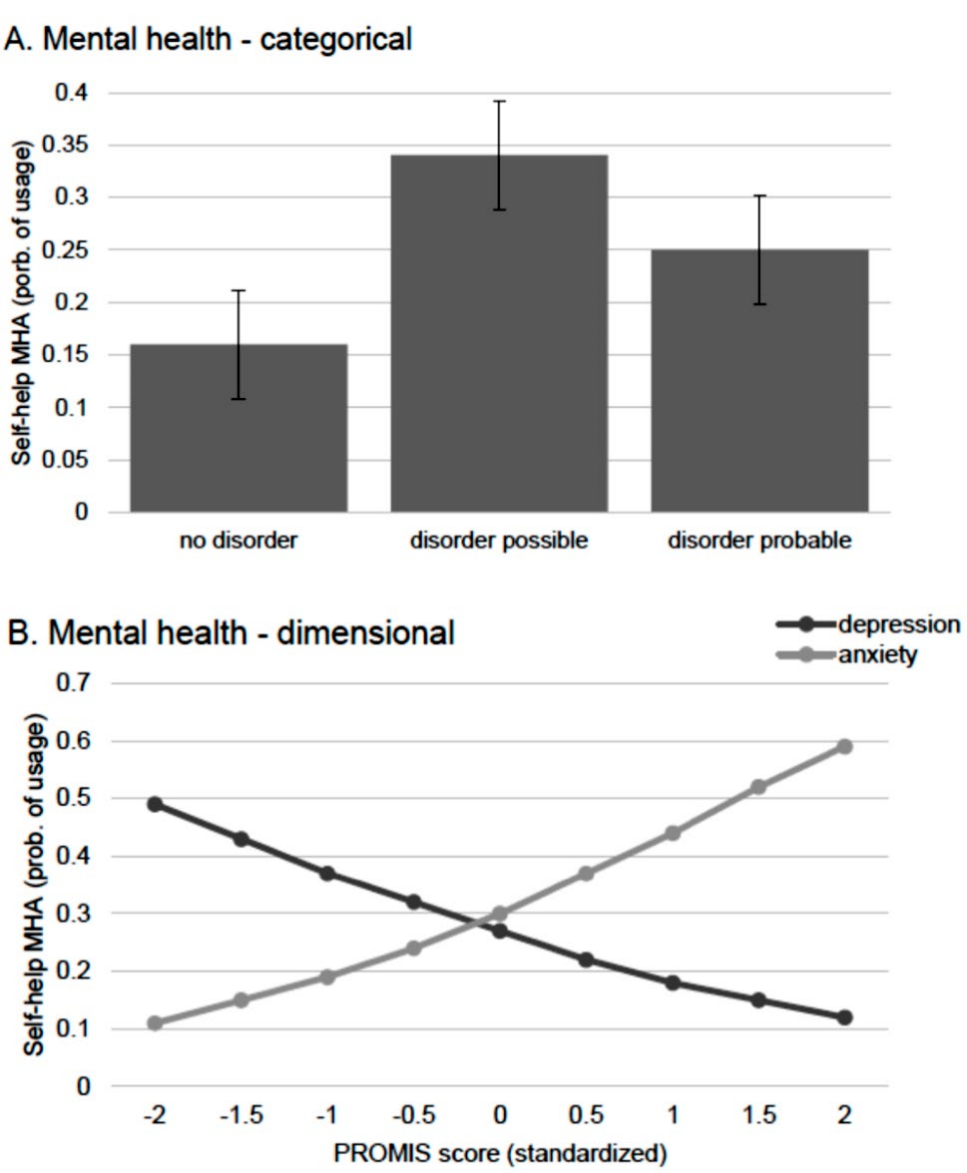
Predictive margins for associations between mental health and usage of self-help MHA. Data are weighted. **(A)** Categorical: no vs. possible vs. probable psychological disorder, assessed via CID-5-S. *n* = 327 users of self-help MHA and *n* = 832 non-users. **(B)** Dimensional: depression and anxiety (*M*, *SE*), assessed via PROMIS. *n* = 327 users of self-help MHA and *n* = 822 non-users

The results of the multiple logistic regression analyses investigating associations between MHA usage and mental health indicators are displayed in Tables 4 and 5. From a categorical point of view, positive screening for any mental disorder in the past 12 months was not associated with any MHA usage. Positive screening for symptoms of any mental disorder only (disorder possible but not probable), however, was positively associated with usage of self-help MHA (OR = 2.82; *p* = .001; see also Fig. 3A). From a dimensional perspective, higher current anxiety was associated with a higher probability of any MHA usage during the past 12 months (OR = 1.61, *p* = .019 for any MHA; OR = 1.87, *p* = .008 for self-help MHA; see also Fig. 3B) and specifically with usage of self-help MHA for anxiety (OR = 2.18, *p* < .001). Higher depression scores were linked to lower likelihood MHA usage for all three outcomes (OR = 0.45, *p* < .001 for any MHA; OR = 0.56, *p* = .005 for self-help MHA; OR = 0.47, *p* < .001 for self-care MHA; see also Fig. 3B). However, higher depression was associated with a higher likelihood of usage of specific self-help MHA for depression (OR = 1.52, *p* = .002). There was no significant association with usage of a moodtracker app. Higher chronic stress was positively associated with usage of any MHA (OR = 1.67, *p* = .003) and usage of self-care MHA (OR = 2.02, *p* < .001).

**Table 4:** Results of multiple regression analyses: mental health indicators predicting usage of MHA

	OR [SE]	CI	*p*	
**binary outcome: MHA usage y/n**				
*Model 1a: mental health - categorical*				
CID-5-S screening:				
disorder possible disorder probable gender age education	1.11 [.34] .77 [.25] .84 [.18] .99 [.01] 1.08 [.28]	[.61; 2.01] [.40; 1.46] [.55; 1.28] [.97; 1.00] [.65; 1.80]	.728 .420 .428 .184 .763	
*Model 1b: mental health - dimensional*				
PROMIS depression PROMIS anxiety TICS stress gender age education	**.45 [.08]** **1.61[.33]** **1.67 [.30]** .97 [.21] 1.00 [01] 1.11 [.28]	**[.31; .63]** **[1.08; 2.39]** **[1.19; 2.40]** [.64; 1.48] [.98; 1.01] [.68; 1.82]	**< .001** **.019** **.003** .901 .661 .669	
**binary outcome: usage self-help y/n**				
*Model 2a: mental health - categorical*				
CID-5-S screening:				
disorder possible disorder probable gender age education	**2.82 [.84]** 1.78 [.59] .88 [.19] .98 [.01] .96 [.26]	**[1.57; 5.07]** [.93; 3.42] [.58; 1.34] [.97; 1.00] [.57; 1.64]	**.001** .081 .558 .054 .889	
*Model 2b: mental health - dimensional*				
PROMIS depression PROMIS anxiety TICS stress gender age education	**.56 [.11]** **1.87 [.44]** 1.38 [.25] 1.02 [.22] .99 [.01] .94 [.24]	**[.38; .84]** **[1.17; 2.97]** [.97; 1.96] [.66; 1.56] [.98; .1.01] [.56; 1.56]	**.005** **.008** .076 .938 .229 .804	
**binary outcome: usage self-care y/n**				
*Model 3a: mental health - categorical*				
CID-5-S screening:				
disorder possible disorder probable gender age education	.94 [.33] .71 [.26] .64 [.16] 1.00 [.01] 1.19 [.36]	[.47; 1.86] [.34; 1.46] [.40; 1.03] [.98; 1.01] [.66; 2.14]	.858 .349 .068 .586 .567	
*Model 3b: mental health - dimensional*				
PROMIS depression PROMIS anxiety TICS stress gender age education	**.47 [.09]** 1.29 [.35] **2.02 [.40]** .76 [.18] 1.00 [.01] 1.20 [.35]	**[.32; .69]** [.76; 2.19] **[1.37; 2.98]** [.47; 1.21] [.99; 1.02] [.68; 2.13]		**<. 001** .342 **< .001** .245 .655 .532

*Notes*. Results of weighted binary logistic regression analyses. OR = odds ratio. *n* = 1,055. PROMIS = Patient-Reported Outcome Measurement System, standardized. TICS = Trier Inventory for Chronic Stress, standardized. CID-5-S = adapted Composite International Diagnostic Screener. Gender is coded 0 = female, 1 = male. Age is given in years. Education is coded 0 = no Abitur (high school level), 1 = Abitur.

**Table 5:** Regression analyses predicting usage of specific MHA categories

	OR [*SE*]	CI	*p*
**binary outcome: usage self-help app depression y/n** *Model 3a: depression*			
PROMIS depression gender age education	**1.52 [.20]** .95 [.35] .99 [.01] .74 [.37]	**[1.17; 1.96]** [.46; 1.98] [.97; 1.01] [.28; 1.95]	**.002** .898 .424 .545
**binary outcome: usage moodtracker y/n** *Model 3b: depression*			
PROMIS depression gender age education	1.26 [.17] .89 [.22] .98 [.01] 1.30 [.36]	[.98; 1.64] [.55; 1.45] [.96; .99] [.76; 2.22]	.075 .645 .010 .339
**binary outcome: usage self-help app anxiety y/n** *Model 4: anxiety*			
PROMIS anxiety gender age education	**2.18 [.35]** 1.16 [.39] .99 [.02] .60 [.28]	**[1.59; 2.99]** [.60; 2.23] [.96; 1.02] [.24; 1.50]	**< .001** .658 .593 .277

*Notes*. Results of weighted binary logistic regression analyses. OR = odds ratio. *n* = 1,055. PROMIS = Patient-Reported Outcome Measurement System, standardized. Gender is coded 0 = female, 1 = male. Age is given in years. Education is coded 0 = no Abitur (high school level), 1 = Abitur.

## Discussion

Our analysis of survey-data of 1,247 individuals revealed that MHA usage is popular among the general population, with over 40%w having used at least one MHA within the last twelve months. Usage of self-care apps was reported more frequently than usage of self-help apps, meditation/mindfulness and relaxation were the most popular categories of MHA. This is in line with previous studies also reporting mindfulness and meditation apps as the most popular MHA [3]. The rather high percentages of non-MHA among the reported apps reveal that people apparently also use features of other apps for mental health purposes. This was especially the case for eating disorders, motivation/inspiration, mood-tracking, and sleeping disorders. This could also explain why the percentage of MHA users we found within the general population is somewhat higher than previously reported numbers regarding usage of health apps in general and MHA in particular [17, 28].

Apart from the finding that MHA-users reported less unemployment compared with non-users, there were no significant sociodemographic differences between the two groups. It appears that MHA usage is equally popular among individuals of different gender, age, and socio-economic status. Therefore, our data indicates that underserviced populations, such as e.g. individuals with low income and/or education, do not show an increased likelihood to be reached by MHA, leaving the potential of MHA to fill this treatment-gap somewhat unfulfilled at the current time.

Perceived helpfulness, fun, and availability were reported as the most common reasons for MHA usage, indicating that a low threshold and a desire for entertainment contributes to the popularity of MHA. Loss of interest as the most common reason for discontinuation of usage however shows that attrition is most likely high and usage itself is not necessarily an indication for consistency. These findings might imply that MHA are perceived as a cheap and easily available opportunity for low-threshold input, but not necessarily as a serious source for self-help and support. Previous research investigating users’ reviews of MHA showed that user-friendly design and the user interface were especially important to users [29]. It could therefore also be speculated that loss of interest could be indicative of poor design of the apps, whereas fun during usage might be the result of user-friendly design with high variety in options and content.

Only ca. 20%w of MHA users reported that they used MHA because they felt their symptoms were too low to require professional treatment and therefore self-help via MHA would suffice. This indicates that the potential of MHA as an early intervention preventing development of more severe symptoms is acknowledged by users, but not a main driver of usage at the current time. MHA-users reported that MHA were too expensive (~ 40%w) and that they have concerns about data security (~ 23%w). These findings are in line with previous research showing high levels of concerns regarding data security [30, 31]. It might therefore be possible that information and awareness about secure MHA which are covered by insurance is still insufficient in the general population.

Many non-users reported that they do not perceive MHA to be helpful (~ 38%w), that they distrust MHA (~ 28%w), have concerns about data security (~ 28%w), and that they would rather solve their problems on their own (~ 23%w). This underlines the lack of sufficient knowledge of e.g. prescription apps or other empirically validated MHA in the general population, which could eventually help to grow trust and willingness to use MHA.

Our results regarding mental health show that a positive screening for any psychological disorder was not associated with MHA usage in general. Symptoms of psychological disorders without impairment however were associated with a higher likelihood of usage of self-help MHA. We can assume that individuals with probable psychological disorders therefore do not perceive MHA as a valid option for self-help. Instead, this option is acknowledged and used by individuals with mental health problems who do not feel impaired (yet). These findings are promising: Individuals with low symptom severity, who might profit from MHA, are more likely to use them than individuals with more severe mental health problems, who might not profit from self-help alone. Individuals with higher anxiety were more likely to use MHA in general and self-help apps for anxiety in particular. Individuals with higher depression also reported more usage of specific self-help apps for depression, but were less likely to generally use MHA. These findings from categorical and dimensional perspectives on mental health indicate that MHA appear to reach their target population (i.e., individuals with mental health problems of low severity) to some extent. Individuals with higher chronic stress reported more usage of MHA in general and self-care MHA in particular. This also indicates that self-care MHA appear to reach at least a part of their target population (i.e., individuals at risk for the development of mental health problems) and could possible contribute to stress-management and prevention of more severe mental health problems.

Because (to our knowledge) no previous research has investigated these topics, it is difficult to discuss these results against the backdrop of previous literature. Our findings regarding relatively high usage of MHA for fun and low-threshold input are in line with previous research reporting frequent use of self-help in general and MHA in particular in the general population [3, 18]. It should also be considered that our data was collected after the pandemic, which might have increased popularity of digital healthcare options. Loss of interest as the most common reason for discontinuation could provide an explanation for the high attrition rates reported by in a review of the topic by Linardon & Fuller-Tyszkiewicz [7]. However, lack of perceived helpfulness and a general distrust might also contribute to low usage as well as high attrition. It is possible that distrust, lack of perceived helpfulness, and subsequent non-usage are the result of the unstructured and confusing app-market and the poor quality of most freely available MHA. Nevertheless, our findings show that individuals with mental health concerns below the threshold of probable psychological disorder tend to use MHA. Individuals with mental health concerns regarding depression, anxiety, and stress also tend to use self-help MHA specifically designed for these issues and individuals with higher stress tend to use self-care MHA. It is promising to see that target populations are reached at least to some extent despite of the barriers reported above.

Previous research that focused not on MHA, but on health apps in general (medication tracking, heartrate monitoring etc.), found app-users to be younger and better educated than non-users [28, 31, 32]. Our findings are not in line with these previous reports, as we could not find significant associations between MHA usage and most sociodemographic characteristics. It could be speculated that health apps for physical wellbeing might require more expertise or knowledge of medical terminology etc., therefore reaching individuals with higher education. MHA on the other hand might be easier to use without specific knowledge of health conditions etc.

It is disappointing that despite the integration of validated prescription MHA into the public health insurance system, this option was only used by 1.5% of MHA-users. Even though this strategy to ensure quality and effectiveness as well as payment channels via insurance is recommended by experts [15, 16], it does not yet seem to transfer into practice. Our findings underline the results of previous research indicating that less than 1% of the population have heard of prescription MHA [30], and that general practitioners (GPs) very rarely inform their patients about prescribable MHA [30], have low knowledge on MHA [33], and especially GPs in rural areas are sceptic and unwilling to prescribe MHA [34]. It appears that GPs need to receive more information and encouragement to reduce skepticism and increase willingness to prescribe MHA.

Several limitations should be considered when interpreting our findings. First, our self-selected sample is not representative of the general population. Especially clickworker-participants might differ from the general population. Even though we employed sample weights to ensure representativeness regarding age and sex, future research should consider stratified sampling in the broader general population to obtain more conclusive results. Second, due to technical problems, a rather large proportion of data on sociodemographic aspects was not available, which further limits representativeness even though missings were random. Third, our results cannot provide information on directionality of associations. A longitudinal study design could enable more detailed research on causality of associations between e.g. MHA-usage and mental health. Fourth, we used a self-developed questionnaire for the assessment of MHA usage instead of established measures. This is a drawback and validated questionnaires, e.g. regarding usability/consumer aspects, might have enriched our data. However, no established instrument was available for our assessment of MHA usage from perspective of help-seeking and usage of healthcare services. Additionally, employment of interviews and qualitative methods could also provide deeper insight. This should be taken into consideration by future studies. And lastly, while we found no association between positive screening for any psychological disorder and MHA usage, future research should investigate this particular population in more detail to provide more insight in differences regarding MHA usage between individuals with vs. without psychological disorders.

## Conclusions

MHA-users cannot be characterized by specific sociodemographic aspects. Self-care apps (especially meditation/mindfulness) are popular in the general population, prescription apps for specific mental health problems on the contrary are currently rarely used. Individuals with higher anxiety, depression, and stress report higher usage of (domain-specific) MHA, which is promising and indicates that target populations can be reached. However, a large part of the general population appears to be skeptical and shows distrust against MHA, indicating that more information is needed. Most likely this is also the case for GPs, who might need encouragement to prescribe MHA.

## Electronic Supplementary Material

Below is the link to the electronic supplementary material.

Supplementary Material 1

## Data Availability

The dataset and materials analyzed during the study are available from the corresponding author upon request. a.
