# Supplementary material for: Characteristic of mental health app usage: a cross-sectional survey in the general population
Source: BMC Public Health. 2024 Nov 12;24:3133. doi: 10.1186/s12889-024-20500-1 (PMC11555976; doi:10.1186/s12889-024-20500-1)
Supplement: Supplementary file 1 — Supplementary Material 1 [file 12889_2024_20500_MOESM1_ESM.docx]

| *Table S1*. Reported usage of MHA by category - not weighted | | | |
| --- | --- | --- | --- |
|  | **no. of users**  **(*N* = 1,247)** | **no. of non-MHA entries** | **no. of excluded entries** |
| *self-help apps:*  moodtracker  self-help depression  self-help anxiety  self-help alcohol/drugs  self-help eating disorders  self-help chronic pain  self-help sleeping disorders  self-help other  *self-care apps:*  meditation/mindfulness  relaxation  motivation/inspiration  *other* | **199 (15.8%)**  98 (7.8%)  88 (7.0%)  49 (3.9%)  44 (3.5%)  65 (5.2%)  **138 (10.9%)**  100 (7.9%)  **245 (19.4%)**  132 (10.5%)  121 (9.6%)  91 (7.2%) | **19 (9.6%)**  4 (4.1%)  4 (4.6%)  1 (2.0%)  1 (2.3%)  2 (3.1%)  **12 (8.7%)**  6 (6.0%)  7 (2.9%)  6 (4.6%)  **10 (8.3%)**  4 (4.4%) | 7 (3.4%)  8 (7.6%)  4 (4.4%)  2 (3.9%)  **11 (20.0%)**  3 (4.4%)  7 (4.8%)  2 (2.0%)  12 (4.7%)  10 (7.0%)  **22 (15.4%)**  **10 (9.9%)** |
| *Notes.* MHA = mental health app. Percentages in the first column are the proportion of all participants. Percentages in the second column are the proportion of non-MHA entries per category. Percentages in the last column are proportions of excluded entries per category. For each variable, the top categories are marked bold. | | | |

| *Table 2*. Sociodemographic characteristics of MHA users and non-users - not weighted | | | |
| --- | --- | --- | --- |
|  | **MHA users (*n* = 511)** | **non-users (*n* = 752)** | **group comparison** |
| age [*M* ± *SD*]  16 - 24 [*n*]  25 - 34 [*n*]  35 - 44 [*n*]  45 - 54 [*n*]  55 - 64 [*n*]  65+ [*n*]  *gender*  female [*n*]  male [*n*]  other [*n*]  *education*  Abitur [*n*]  no Abitur [*n*]  *employment*  employed [*n*]  unemployed [*n*]  *income*  < 500€/month [*n*]  500 - 1,000€/month [*n*]  1,000 - 2,000€/month [*n*]  2,000 - 3,500€/month [*n*]  3,500-5,000€/month [*n*]  > 5,000€/month [*n*]  *living area*  rural [*n*]  town [*n*]  city [*n*]  big city [*n*] | 33.00 ± 11.25  139 (27.2%)  197 (38.6%)  89 (17.4%)  53 (10.4%)  26 (5.1%)  7 (1.4 %)  299 (58.5%)  205 (40.1%)  7 (1.4%)  371 (78.9%)  99 (21.6%)  379 (80.6%)  91 (19.4%)  21 (4.5%)  56 (11.9%)  100 (21.3%)  158 (33.6%)  92 (19.6%)  43 (9.2%)  78 (15.3%)  98 (19.2%)  131 (25.6%)  204 (39.9%) | 32.25 ± 11.69  216 (28.7%)  308 (41.0%)  116 (15.4%)  52 (6.9%)  48 (6.4%)  12 (1.6%)  436 (58.0%)  307 (40.8%)  9 (1.2%)  491 (82.0%)  108 (18.0%)  443 (74.0%)  156 (26.0%)  26 (4.3%)  81 (13.5%)  145 (24.2%)  171 (28.6%)  124 (20.7%)  52 (8.7%)  137 (18.2%)  105 (14.0%)  171 (22.7%)  339 (45.1%) | *F* = 1.32, *p* = .251  χ² = .12, *p* = .940  χ² = 1.55, *p* = .213  **χ² = 6.62, *p* = .010**  χ² = 3.96, *p* = .556  **χ² = 9.65, *p* = .022** |
| *Notes*. MHA = mental health application. Age is given in years. Abitur = high school level education. Available data for education, employment, income, and living area from *n* = 470 MHA users and *n* = 599 non-users. | | | |

| *Table 3*. Mental health characteristics of MHA users and non-users - not weighted | | | | | |  |
| --- | --- | --- | --- | --- | --- | --- |
|  | **MHA users  (*n* = 511)** | **non-users  (*n* = 752)** | | **group comparison** | |  |
|  |  |  | |  | |  |
| *categorical*  CID-5-S screening: [*n* (%)]  no disorder  disorder possible  disorder probable  *dimensional*  PROMIS depression [*M* ± *SD*]  PROMIS anxiety [*M* ± *SD*]  TICS stress [*M* ± *SD*] | 76  253 (51.2)  **165 (33.4)**  2.54 ± .96  **2.49 ± .93**  18.62 ± 10.77 | | 318 (46.8)  **271 (39.9)**  2.55 ± 1.00  **2.35 ± .93**  17.46 ± 11.10 | | χ² = 2.27, *p* = .132  **χ² = 5.10, *p* = .024**  *F* = .02, *p* = .888  ***F* = 5.78, *p* = .016**  *F* = 3.17, *p* = .075 | |
| *Notes*. Results of group comparison tests. MHA = mental health application. Depression and anxiety are given as the Patient Reported Outcome Measurement Systems (PROMIS) mean score, possible range 1-5, available data from *n* = 494 MHA users and *n* = 670 non-users. Stress is given as the Trier Inventory for Chronic Stress (TICS) sum score, possible range 0-48, available data from *n* = 485 MHA users and *n* = 646 non-users. Possible and likely disorders are given as determined by the adapted Composite International Diagnostic – Screener (CID-5-S), available data from *n* = 494 MHA users and *n* = 680 non-users. | | | | | |  |

| *Table 4.* Results of multiple regression analyses: mental health indicators predicting usage of MHA - not weighted | | | | |  |
| --- | --- | --- | --- | --- | --- |
|  | **OR [SE]** | **CI** | ***p*** | |  |
| **binary outcome: MHA usage y/n** | | | | |  |
| *Model 1a: mental health - categorical* | | | | |  |
| CID-5-S screening:  disorder possible  disorder probable  gender  age  education | 1.11 [.34]  .77 [.25]  .84 [.18]  .99 [.01]  1.08 [.28] | .  [.61; 2.01]  [.40; 1.46]  [.55; 1.28]  [.97; 1.00]  [.65; 1.80] | .728  .420  .428  .184  .763 | |  |
| *Model 1b: mental health - dimensional* | | | | |  |
| PROMIS depression  PROMIS anxiety  TICS stress  gender  age  education | **.45 [.08]**  **1.61[.33]**  **1.67 [.30]**  .97 [.21]  1.00 [01]  1.11 [.28] | **[.31; .63]**  **[1.08; 2.39]**  **[1.19; 2.40]**  [.64; 1.48]  [.98; 1.01]  [.68; 1.82] | **< .001**  **.019**  **.003**  .901  .661  .669 | |  |
| **binary outcome: usage self-help y/n** | | | | |  |
| *Model 2a: mental health - categorical* | | | | |  |
| CID-5-S screening:  disorder possible  disorder probable  gender  age  education | **2.82 [.84]**  1.78 [.59]  .88 [.19]  .98 [.01]  .96 [.26] | **[1.57; 5.07]**  [.93; 3.42]  [.58; 1.34]  [.97; 1.00]  [.57; 1.64] | **.001**  .081  .558  .054  .889 | |  |
| *Model 2b: mental health - dimensional* | | | | |  |
| PROMIS depression  PROMIS anxiety  TICS stress  gender  age  education | **.56 [.11]**  **1.87 [.44]**  1.38 [.25]  1.02 [.22]  .99 [.01]  .94 [.24] | **[.38; .84]**  **[1.17; 2.97]**  [.97; 1.96]  [.66; 1.56]  [.98; .1.01]  [.56; 1.56] | **.005**  **.008**  .076  .938  .229  .804 | |  |
| **binary outcome: usage self-care y/n** | | | | |  |
| *Model 3a: mental health - categorical* | | | | |  |
| CID-5-S screening:  disorder possible  disorder probable  gender  age  education | .94 [.33]  .71 [.26]  .64 [.16]  1.00 [.01]  1.19 [.36] | [.47; 1.86]  [.34; 1.46]  [.40; 1.03]  [.98; 1.01]  [.66; 2.14] | .858  .349  .068  .586  .567 | |  |
| *Model 3b: mental health - dimensional* | | | | |  |
| PROMIS depression  PROMIS anxiety  TICS stress  gender  age  education | **.47 [.09]**  1.29 [.35]  **2.02 [.40]**  .76 [.18]  1.00 [.01]  1.20 [.35] | **[.32; .69]**  [.76; 2.19]  **[1.37; 2.98]**  [.47; 1.21]  [.99; 1.02]  [.68; 2.13] | | **<. 001**  .342  **< .001**  .245  .655  .532 | |
| *Notes*. Results of binary logistic regression analyses. OR = odds ratio. *n* = 1,055. PROMIS = Patient-Reported Outcome Measurement System, standardized. TICS = Trier Inventory for Chronic Stress, standardized. CID-5-S = adapted Composite International Diagnostic Screener. Gender is coded 0 = female, 1 = male. Age is given in years. Education is coded 0 = no Abitur (high school level), 1 = Abitur. | | | | |  |
